# Twenty-Two Months of Syndromic Multiplex-PCR Testing for Acute Infections in a Southern Italian Hospital: Pathogen Epidemiology, Diagnostic Appropriateness and the Cost of Negative Results

**DOI:** 10.3390/microorganisms14071574

**Published:** 2026-07-19

**Authors:** Daniela Chirizzi, Angela Spedicato, Gabriele Bianco, Laura Lupo, Ilaria Serafini, Daniele Pisanò, Maria Rita Orsi, Giovanni Moschettini, Angelo Sorge, Rosanna Bruno, Camilla Panico, Silvana Arnesano, Antonella Simone, Luciana Corciulo, Antonella Pico, Claudia Pagano, Letizia Fulceri, Giulia Apruzzi, Anna De Filippis, Massimiliano Galdiero, Francesco Broccolo

**Affiliations:** 1 UOSD Microbiology and Virology, “Vito Fazzi” Hospital, ASL Lecce, 73100 Lecce, Italyangelo.sorge@asl.lecce.it (A.S.);; 2 Department of Experimental Medicine, University of Salento, 73100 Lecce, Italy; 3 UOC Hospital Pharmacy, “Vito Fazzi” Hospital, ASL Lecce, 73100 Lecce, Italy; 4 Department of Woman, Child and General and Specialized Surgery, University of Campania “Luigi Vanvitelli”, 80138 Naples, Italy; anna.defilippis@unicampania.it (A.D.F.);; 5 UOC Virology and Microbiology, University Hospital of the University of Campania “Luigi Vanvitelli”, 80138 Naples, Italy

**Keywords:** syndromic multiplex PCR, BioFire FilmArray, diagnostic stewardship, meningitis/encephalitis panel, respiratory pathogen panel, pneumonia panel, antimicrobial resistance, influenza A subtyping, avian influenza H5N1, diagnostic appropriateness

## Abstract

Syndromic multiplex-PCR panels deliver rapid, comprehensive detection of pathogens and resistance determinants in acute infections, but their unrestricted use can generate a high proportion of negative results with substantial economic and stewardship implications. We retrospectively analysed all BioFire FilmArray meningitis/encephalitis (ME), upper-respiratory (RP2.1), pneumonia (PN) and gastrointestinal (GI) determinations performed at the UOSD Microbiology and Virology of P.O. “Vito Fazzi”, ASL Lecce (Apulia, Italy) between 1 July 2024 and 30 April 2026. Repeat determinations from the same patient with the same panel were identified in the laboratory information system and removed before any analysis, so that each determination analysed corresponds to a distinct diagnostic episode. Across 5381 determinations (CNS, *n* = 761; upper respiratory, *n* = 2601; lower respiratory, *n* = 399; gastrointestinal, *n* = 1620), 59.3% were negative for every target, with a steep appropriateness gradient: 90.0% negativity for the ME panel and 77.1% for the gastrointestinal panel versus 44.2% for the upper-respiratory and 26.6% for the pneumonia panel. CNS positives were predominantly viral (75%), led by enterovirus; *Streptococcus pneumoniae* was the only consistent bacterial agent. Human rhinovirus/enterovirus dominated the respiratory ecology; influenza A was almost entirely H3 and H1N1pdm09, but 8.1% of influenza-A–positive specimens were equivocal or non-subtypeable and were never referred for sequencing, an avoidable surveillance blind spot for novel/zoonotic (avian) influenza. Pneumonia-panel resistance markers (mecA/C–MREJ, CTX-M, KPC, NDM) clustered in Enterobacterales co-infections. The gastrointestinal panel was dominated by diarrhoeagenic *Escherichia coli* pathotypes (chiefly EPEC and EAEC) and *Clostridioides difficile* toxin, its 77% negativity identifying a second over-utilised stream. We argue for CSF-pleocytosis gating, tiered/reflex respiratory algorithms, gastrointestinal-panel gating to community-onset diarrhoea, and mandatory reflex sequencing of unsubtypeable influenza A.

## 1. Introduction

Syndromic multiplex polymerase chain reaction (PCR) panels have transformed the diagnosis of acute infections by simultaneously interrogating a fixed list of pathogens (and, for some panels, antimicrobial-resistance determinants) directly from a clinical specimen within approximately one to two hours [1,2]. In the diagnosis of central nervous system (CNS), upper- and lower-respiratory infections, this sample-to-answer approach compresses a diagnostic work-up that traditionally required culture, serology and reflex molecular assays into a single, low-hands-on-time test, with demonstrable reductions in time-to-result and, in several studies, earlier and more appropriate antimicrobial and antiviral decisions [1,2,3].

These advantages, however, are inseparable from two structural drawbacks. First, because the panels are fast, easy to order and broad in scope, they are prone to over-utilisation: when applied without pre-test clinical or laboratory gating, a large fraction of determinations return negative for every target, consuming reagents and analyser capacity without altering management [1,2,4]. The clearest example is the BioFire FilmArray ME panel, for which restricting testing to patients with cerebrospinal fluid (CSF) pleocytosis reduces utilisation by roughly 40% while increasing diagnostic yield by more than 60% [4]. Second, syndromic panels detect what they are designed to detect and nothing more: their built-in influenza-A subtyping resolves only seasonal human subtypes (H1, H1N1pdm09, H3), so a novel or zoonotic strain, most pressingly the highly pathogenic avian influenza A (H5N1) lineage now causing mammalian and sporadic human infections, can be flagged as “influenza A, no subtype detected” or “equivocal” and, in the absence of reflex referral and sequencing, escape recognition entirely [5,6,7].

The province of Lecce, in the Salento peninsula at the south-eastern tip of Apulia, is a defined catchment served by a single hub microbiology and virology laboratory at P.O. “Vito Fazzi”. It combines a Mediterranean climate, intense seasonal tourism, an ageing resident population, and an agricultural landscape with backyard poultry and coastal wetlands along a major migratory flyway, features that make it both a representative setting for studying real-world syndromic-panel use and a sentinel territory for zoonotic respiratory viruses. Here we describe twenty-two months of consecutive ME, upper-respiratory, pneumonia and gastrointestinal panel determinations, characterise the local epidemiology of acute infections, quantify the appropriateness gradient across clinical settings, estimate the economic footprint of negative results, and highlight the avian influenza surveillance gap created by unsubtyped influenza A. We frame the findings against international experience with the same platforms to propose targeted, stewardship-based diagnostic pathways.

## 2. Materials and Methods

### 2.1. Setting and Study Period

This single-centre, retrospective, laboratory-based study was conducted at the UOSD Microbiologia e Virologia Universitaria of P.O. “Vito Fazzi”, ASL Lecce, the reference microbiology laboratory for the Lecce health district (Apulia, Italy). All consecutive syndromic multiplex-PCR determinations performed on acute-care specimens between 1 July 2024 and 30 April 2026 were included. Three reporting periods are presented throughout: the second half of 2024 (July–December), the full calendar year 2025, and the first four months of 2026 (January–April).

### 2.2. Assays

Four BioFire FilmArray panels (BioFire Diagnostics, a bioMérieux company, Salt Lake City, UT, USA; bioMérieux SA, Marcy-l’Étoile, France) were used according to the manufacturer’s instructions on FilmArray 2.0 and FilmArray Torch systems (BioFire Diagnostics, Salt Lake City, UT, USA): (i) the Meningitis/Encephalitis (ME) panel on CSF (14 targets: *Escherichia coli* K1, *Haemophilus influenzae*, *Listeria monocytogenes*, *Neisseria meningitidis*, *Streptococcus agalactiae*, *Streptococcus pneumoniae*, cytomegalovirus, enterovirus, herpes simplex virus 1 and 2, human herpesvirus 6, human parechovirus, varicella-zoster virus and *Cryptococcus neoformans/gattii*); (ii) the Respiratory 2.1 (RP2.1) panel for upper-respiratory specimens (viral targets plus *Bordetella* spp. and *Mycoplasma pneumoniae*), which additionally reports influenza-A subtyping (H1, H1-2009/H1N1pdm09, H3); and (iii) the Pneumonia (PN) panel on lower-respiratory specimens, providing semi-quantitative detection of 15 typical bacteria, atypical bacteria, eight viruses and seven antimicrobial-resistance markers (mecA/C and MREJ, CTX-M, KPC, NDM, OXA-48-like, VIM, IMP) [8,9]; and (iv) the Gastrointestinal (GI) panel on stool specimens, providing qualitative detection of 22 enteric targets: bacteria (*Campylobacter* spp., *Clostridioides difficile* toxin A/B, *Plesiomonas shigelloides*, *Salmonella* spp., *Vibrio* spp. and *Vibrio cholerae*, *Yersinia enterocolitica*, and the diarrhoeagenic *Escherichia coli* pathotypes EAEC, EPEC, ETEC [lt/st] and STEC [stx1/stx2, including *E. coli* O157], plus *Shigella*/enteroinvasive *E. coli*), parasites (*Cryptosporidium* spp., *Cyclospora cayetanensis*, *Entamoeba histolytica*, and *Giardia lamblia*) and viruses (adenovirus F40/41, astrovirus, norovirus GI/GII, rotavirus A and sapovirus) [10]. All panel targets are detected and reported at species or pathotype level only, without capsular characterisation: on the ME panel the Neisseria meningitidis assay detects encapsulated strains (serogroups A, B, C, W, X and Y) but does not discriminate among them, the Haemophilus influenzae assay detects both typeable (serotypes a–f) and non-typeable strains without assigning a serotype, and the Streptococcus pneumoniae assay does not resolve capsular serotypes. Accordingly, no serogrouping or serotyping of *N. meningitidis*, *H. influenzae* or *S. pneumoniae* was performed in this study, which rests exclusively on molecular detection directly from the clinical specimen; culture isolates suitable for capsular typing were not available for the determinations analysed here, and no data on vaccine-type distribution can therefore be derived from this series. No additional, off-panel testing for agents not represented on the four target lists (for example, Toscana virus, West Nile virus or other arboviruses in cerebrospinal fluid) was performed during the study period.

### 2.3. Data Extraction and Analysis

Aggregate, fully de-identified counts of determinations, single-pathogen positives, co-infections (≥2 targets in the same specimen), all-negative results, per-target detections and resistance-gene detections were extracted from the laboratory information system (DNLab, Dedalus S.p.A., Florence, Italy) for each panel and period. Repeat determinations performed on the same patient with the same panel were traced through the laboratory information system and duplicate records were removed a priori, before any analysis: no patient therefore contributes more than one determination per panel and per clinical episode to the denominators reported below, and the counts presented are counts of distinct diagnostic episodes rather than of cartridges consumed for a given patient. Positivity and negativity were expressed as a percentage of determinations; per-target prevalence was expressed as a percentage of total determinations for the relevant panel, so that, in the presence of co-infections, the sum of per-target prevalences may exceed the proportion of positive specimens. The influenza-A subtyping summary is reported over the subtyping-evaluable subset. Because only aggregate laboratory data were available, appropriateness was inferred at the level of diagnostic yield by clinical setting rather than from individual ordering indications. Appropriateness was accordingly defined operationally, and at the level of the testing stream rather than of the single request, as the diagnostic yield obtained in a given clinical setting, that is, the proportion of determinations returning at least one detected target, taken as a proxy for the pre-test probability of the population actually submitted to testing: a stream in which the large majority of determinations return negative for every target is one in which the panel is being applied beyond the population in whom it can plausibly yield an actionable result, and is on that basis considered over-utilised. This definition is deliberately conservative and does not classify any individual request as appropriate or inappropriate, a judgement that would require the clinical indication, the cerebrospinal fluid cell count, the host status, and the severity of presentation, none of which is retrievable from aggregate laboratory data. Descriptive statistics were used to summarise activity, yield and cost. Categorical comparisons between panels (all-negative versus positive determinations; co-infections as a share of positives) were tested with Pearson’s chi-square test on the corresponding contingency tables, with Fisher’s exact test applied where any expected cell count fell below five; all tests were two-sided, and a *p*-value below 0.05 was considered significant. Analyses were performed in Python 3.11 (Python Software Foundation, Wilmington, DE, USA) using the SciPy 1.11 and pandas 2.2 libraries (open-source software); figures were generated with Matplotlib 3.8 (open-source software). No individual patient data were analysed.

## 3. Results

### 3.1. Overall Activity and the Appropriateness Gradient

During the 22-month period, 5381 syndromic determinations were performed: 761 ME, 2601 upper-respiratory, 399 pneumonia and 1620 gastrointestinal panels. Overall, 3190 determinations (59.3%) were negative for every target. Negativity, however, varied markedly by clinical setting, defining a clear appropriateness gradient (Table 1): the ME panel returned no detectable pathogen in 90.0% of CSF samples and the gastrointestinal panel in 77.1% of stool samples, compared with 44.2% for upper-respiratory and only 26.6% for pneumonia specimens. The difference in negativity across the four panels was highly significant (χ^2^ = 932.4, df = 3, *p* < 0.001) and remained so in every pairwise comparison, including those between the adjacent extremes of the gradient (ME versus GI, χ^2^ = 56.6, *p* < 0.001; upper-respiratory versus pneumonia, χ^2^ = 44.3, *p* < 0.001). The proportion of positives represented by co-infections followed an inverse gradient (0% for CNS, 12.9% for gastrointestinal, 18.5% for upper-respiratory, and 48.8% for lower-respiratory positives), consistent with a polymicrobial lower-airway ecology and an essentially mono-aetiological CNS picture. This between-panel difference in the co-infection share of positives was itself highly significant (χ^2^ = 177.2, df = 3, *p* < 0.001).

### 3.2. Meningitis/Encephalitis Panel: Viral Predominance and Minimal Bacterial Yield

Of 76 ME-panel positives, 57 (75%) were viral and 19 (25%) bacterial; no fungal target (*Cryptococcus*), no *E. coli* K1, no cytomegalovirus, and no human parechovirus were detected, and no co-infection occurred in any CSF specimen (Table 2). Enterovirus was the single most frequent agent (27 cases; 35.5% of all positives), followed by herpes simplex virus 2 and varicella-zoster virus (nine each), herpes simplex virus 1 and human herpesvirus 6 (6 each). On the bacterial side, *S. pneumoniae* was the only consistently recovered pyogenic agent (14 cases across all periods), while *S. agalactiae* (2), *H. influenzae* (1), *L. monocytogenes* (1) and *N. meningitidis* (1) appeared sporadically and almost exclusively during the higher-volume 2025 period.

**Table 2:** Meningitis/encephalitis (ME) panel: Detections by pathogen and reporting period (CSF, *n* = 761; 76 positive determinations, no co-infections).

Target	2024 H2	2025	2026	Total
**Bacteria**				
*Streptococcus pneumoniae*	4	8	2	14
*Streptococcus agalactiae*	0	2	0	2
*Haemophilus influenzae*	0	1	0	1
*Listeria monocytogenes*	0	1	0	1
*Neisseria meningitidis*	0	1	0	1
*Escherichia coli* K1	0	0	0	0
**Viruses**				
Enterovirus	9	14	4	27
Herpes simplex virus 2	3	4	2	9
Varicella-zoster virus	3	5	1	9
Herpes simplex virus 1	1	4	1	6
Human herpesvirus 6	3	2	1	6
Cytomegalovirus	0	0	0	0
Human parechovirus	0	0	0	0
**Fungi**				
*Cryptococcus neoformans/gattii*	0	0	0	0
**Total positive**	23	42	11	76

### 3.3. Upper-Respiratory Panel: Rhinovirus/Enterovirus Dominance

**Figure 1:**
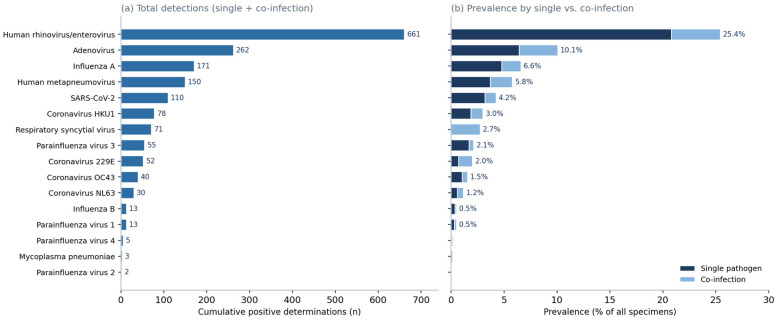
Upper-respiratory panel (RP2.1), July 2024–April 2026 (*n* = 2601). Cumulative positive determinations (**a**) and per-pathogen prevalence as a percentage of all specimens, stratified into single-pathogen and co-infection contributions (**b**). Note that the respiratory syncytial virus was detected exclusively within co-infections.

Across 2601 upper-respiratory determinations, 1451 (55.8%) were positive, and 268 positives (18.5%) were co-infections. The cumulative sum of per-target prevalences reached 65.7% of all specimens. Human rhinovirus/enterovirus was overwhelmingly dominant (661 detections; 25.4% of all specimens), more than double the second-ranked adenovirus (262; 10.1%), and followed by influenza A (171; 6.6%), human metapneumovirus (150; 5.8%) and SARS-CoV-2 (110; 4.2%) (Table 3, Figure 1). The first-ranked agent alone accounted for 25.4% of specimens, the top three for 42.1%, and the top five for 52.1%. Seasonal coronaviruses (HKU1, 229E, OC43, and NL63) together contributed a further 200 detections. Two findings deserve emphasis: respiratory syncytial virus (RSV) was detected 71 times but never as a single pathogen (appearing exclusively within co-infections), and *Bordetella* spp. and MERS-CoV were never detected. The RSV co-detections involved most frequently human metapneumovirus and human rhinovirus/enterovirus. Because the extraction returned aggregate counts without age, host-status, or week-of-collection fields, this pattern could not be stratified by paediatric or immunocompromised status or by sampling season, and is discussed below as a hypothesis-generating observation on the composition of the tested population rather than as an epidemiological estimate (Section 4.1 and Section 4.7).

**Table 3:** Upper-respiratory (RP2.1) panel: Per-target detections (single + co-infection) and prevalence over all determinations (*n* = 2601), ranked.

Target	Detections	% of N	Note
Human rhinovirus/enterovirus	661	25.4	Most prevalent; year-round
Adenovirus	262	10.1	Frequent co-infections
Influenza A	171	6.6	H3 > H1N1pdm09 (Table 4)
Human metapneumovirus	150	5.8	Often with RSV
SARS-CoV-2	110	4.2	Endemic/seasonal
Coronavirus HKU1	78	3.0	
Respiratory syncytial virus	71	2.7	Only in co-infection
Parainfluenza virus 3	55	2.1	
Coronavirus 229E	52	2.0	
Coronavirus OC43	40	1.5	
Coronavirus NL63	30	1.2	
Influenza B	13	0.5	
Parainfluenza virus 1	13	0.5	
Parainfluenza virus 4	5	0.2	
*Mycoplasma pneumoniae*	3	0.1	Only in co-infection
Parainfluenza virus 2	2	0.1	
MERS-CoV/*Bordetella* spp.	0	0	Never detected
Cumulative (sum of detections)	1716	65.7	

Cumulative (sum of detections), sum of the per-target detections; because a single specimen may contain more than one target, this sum exceeds the number of positive determinations and the corresponding percentage exceeds the overall positivity rate of the panel.

### 3.4. Influenza-A Subtyping and the Avian Influenza Blind Spot

**Figure 2:**
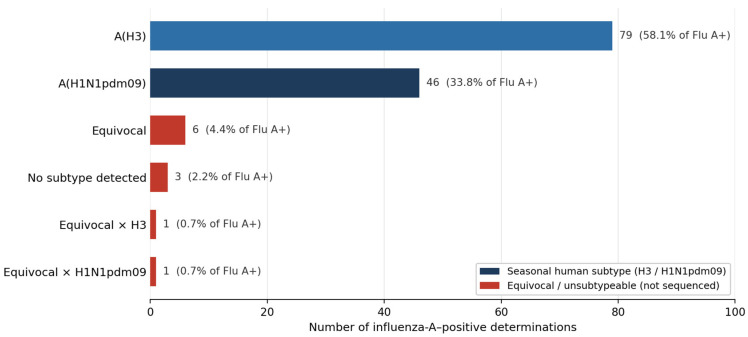
Influenza-A subtype distribution, July 2024–April 2026 (136 influenza-A–positive determinations/2495 specimens). The categories shown in red (equivocal and “no subtype detected”; 11 determinations, 8.1% of influenza-A positives) were not referred for reflex sequencing and constitute the avian influenza surveillance blind spot discussed in the text. Blue bars denote influenza-A determinations for which a seasonal human subtype was resolved (H3, H1N1pdm09); red bars denote equivocal or non-subtypeable results. The two shades of blue carry no additional meaning and have been harmonised into a single blue in the final version of the figure.

Within the subtyping-evaluable subset (136 influenza-A–positive determinations across 2495 specimens; 5.5% positivity), the panel resolved a seasonal-subtype profile dominated by A(H3) (79; 58.1% of influenza-A positives) and A(H1N1pdm09) (46; 33.8%) (Table 4, Figure 2). Critically, 11 influenza-A–positive determinations (8.1%) were not cleanly subtyped: 6 “equivocal”, 3 “no subtype detected”, and 1 each of “equivocal × H3” and “equivocal × H1N1pdm09”. None of these specimens underwent reflex sequencing. The eleven determinations were distributed across both influenza seasons covered by the study, broadly in proportion to the underlying seasonal influenza-A activity (four in the 2024–2025 season and seven in the 2025–2026 season), and all of them fell inside the weeks of documented seasonal influenza-A circulation rather than outside the epidemic window; all originated from hospital-based requests within the ASL Lecce catchment (emergency department and inpatient wards), and none carried a recorded avian, poultry or other animal exposure on the request form. Beyond this, no structured epidemiological exposure history could be retrieved, which is itself part of the problem we describe: in the absence of a standing reflex rule, neither the laboratory nor the requesting clinician is prompted to collect, or to act upon, exposure information at the moment a subtype fails to resolve. Because the panel’s subtyping chemistry targets only currently circulating human subtypes, this unresolved category is precisely the pattern that a novel or zoonotic influenza A, including highly pathogenic avian A(H5N1), would produce; without onward referral, such a case would be reported simply as “influenza A” and would not be recognised.

**Table 4:** Influenza-A subtyping summary (subtyping-evaluable subset; 136 influenza-A positives/2495 specimens). Categories marked with an asterisk constitute the unsubtypeable cluster that was not sequenced.

Subtype/Outcome	*n*	% of Influenza A+	% of Specimens
A (H3)	79	58.1	3.17
A (H1N1pdm09)	46	33.8	1.84
Equivocal *	6	4.4	0.24
No subtype detected *	3	2.2	0.12
Equivocal × H3 *	1	0.7	0.04
Equivocal × H1N1pdm09 *	1	0.7	0.04
Unsubtypeable subset (not sequenced) *	11	8.1	0.44
Total influenza A+	136	100	5.5

Influenza A+, influenza-A-positive determinations; % of influenza A+, percentage over the 136 influenza-A-positive determinations; % of specimens, percentage over the 2495 subtyping-evaluable specimens. * Equivocal and “no subtype detected” results require reflex sequencing; none were referred during the study period.

### 3.5. Pneumonia Panel: Polymicrobial Ecology and Concentrated Resistance

**Figure 3:**
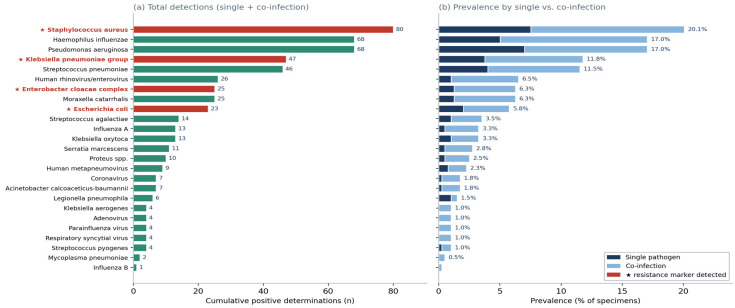
Pneumonia panel, July 2024–April 2026 (*n* = 399). Cumulative positive determinations (**a**) and prevalence as a percentage of specimens (**b**), with single-pathogen and co-infection contributions; ★ denotes organisms for which a resistance marker (mecA/C–MREJ/MRSA, CTX-M, KPC or NDM) was detected. In panel (**a**), red bars and the star symbol identify the organisms for which at least one resistance marker was detected; green bars identify all the other panel targets. In panel (**b**), dark blue denotes the single-pathogen contribution and light blue the co-infection contribution to the prevalence of each target.

The pneumonia panel had the highest yield and the highest co-infection burden: of 293 positive specimens, 143 (48.8%) were polymicrobial. *Staphylococcus aureus* was the single most frequent organism (80 detections; 20.1% of specimens), followed by *Haemophilus influenzae* and *Pseudomonas aeruginosa* (68 each; 17.0%), the *Klebsiella pneumoniae* group (47; 11.8%) and *S. pneumoniae* (46; 11.5%); the top three organisms accounted for 54.1% and the top five for 77.4% of specimens (Table 5, Figure 3). Resistance markers were concentrated rather than diffuse: mecA/C–MREJ (methicillin-resistant *S. aureus*, MRSA) was the most frequent determinant (9 determinations: 2 in 2024, 5 in 2025, 2 in 2026), while the β-lactamase/carbapenemase markers CTX-M, KPC and NDM were detected almost exclusively within severe Enterobacterales co-infections (4 determinations involving the *K. pneumoniae* group and *E. coli*; 1 in 2024, 2 in 2025, 1 in 2026), including a single NDM-positive *K. pneumoniae* in 2026. The remaining carbapenemase targets (IMP, OXA-48-like, and VIM) were never detected.

**Table 5:** Pneumonia (PN) panel: Leading targets (single + co-infection) over all determinations (*n* = 399) and associated resistance markers.

Target	Detections	% of N	Resistance Marker(s)
*Staphylococcus aureus*	80	20.1	mecA/C–MREJ (MRSA), 9 cases
*Haemophilus influenzae*	68	17.0	—
*Pseudomonas aeruginosa*	68	17.0	—
*Klebsiella pneumoniae* group	47	11.8	KPC, CTX-M, NDM
*Streptococcus pneumoniae*	46	11.5	—
Human rhinovirus/enterovirus	26	6.5	—
*Enterobacter cloacae* complex	25	6.3	CTX-M (in co-infection)
*Moraxella catarrhalis*	25	6.3	—
*Escherichia coli*	23	5.8	CTX-M
*Streptococcus agalactiae*	14	3.5	—
Influenza A/*Klebsiella oxytoca*	13/13	3.3	—
*Serratia marcescens*/*Proteus* spp.	11/10	2.8/2.5	—
Other targets (each < 2.5%)	≤9	—	hMPV, coronavirus, *Acinetobacter*, *Legionella*, etc.
IMP/OXA-48-like/VIM	0	0	Never detected

### 3.6. Gastrointestinal Panel: Diarrhoeagenic E. coli Dominance and a High Negativity Rate

The gastrointestinal panel was the second-highest-volume stream (1620 stool determinations) and, after the ME panel, the least likely to yield a pathogen: 1249 specimens (77.1%) were negative for every target, and only 371 (22.9%) were positive, of which 48 (12.9%) were polymicrobial (Table 6). The 371 positive determinations generated 420 pathogen detections, overwhelmingly bacterial (354; 84.3%), with viruses (60; 14.3%) and parasites (6; 1.4%) contributing little. The dominant finding was the diarrhoeagenic *Escherichia coli* group (EPEC, EAEC, ETEC, STEC and *Shigella*/enteroinvasive *E. coli*), which together accounted for 192 detections (45.7% of all detections), led by enteropathogenic *E. coli* (EPEC; 103; 24.5%) and enteroaggregative *E. coli* (EAEC; 58; 13.8%). *Clostridioides difficile* toxin A/B was the second most frequent target overall (88; 21.0%), followed by *Salmonella* spp. (36; 8.6%), norovirus GI/GII (34; 8.1%) and *Campylobacter* spp. (33; 7.9%). Norovirus was the leading viral agent, with rotavirus A, adenovirus F40/41, sapovirus and astrovirus detected sporadically; the parasitic yield was minimal (*Giardia lamblia* 4, *Cryptosporidium* spp. 2), while *Vibrio* spp., *Vibrio cholerae*, *E. coli* O157, *Cyclospora cayetanensis* and *Entamoeba histolytica* were never detected. This profile was stable across the three reporting periods.

**Table 6:** Gastrointestinal (GI) panel: Detections by target (single-pathogen + co-infection) over all determinations (stool, *n* = 1620), ranked by total detections.

Target	Single-Pathogen	Co-Infection	Total Detections (%)
Enteropathogenic *E. coli* (EPEC)	77	26	103 (24.5)
*Clostridioides difficile* toxin A/B	74	14	88 (21.0)
Enteroaggregative *E. coli* (EAEC)	40	18	58 (13.8)
*Salmonella* spp.	30	6	36 (8.6)
Norovirus GI/GII	27	7	34 (8.1)
*Campylobacter* spp.	26	7	33 (7.9)
Shiga-toxin *E. coli* (STEC) stx1/stx2	12	5	17 (4.0)
Adenovirus F40/41	8	2	10 (2.4)
Enterotoxigenic *E. coli* (ETEC) lt/st	5	4	9 (2.1)
Rotavirus A	7	1	8 (1.9)
*Shigella*/enteroinvasive *E. coli* (EIEC)	2	3	5 (1.2)
Sapovirus	4	1	5 (1.2)
*Yersinia enterocolitica*	4	0	4 (1.0)
*Giardia lamblia*	4	0	4 (1.0)
Astrovirus	2	1	3 (0.7)
*Cryptosporidium* spp.	0	2	2 (0.5)
*Plesiomonas shigelloides*	1	0	1
Total/all targets	323	97	420

Stool specimens, 1 July 2024–30 April 2026 (*n* = 1620). Positive determinations: 371 (22.9%); negative: 1249 (77.1%); co-infections: 48 patients (97 of 420 detections). Diarrhoeagenic *E. coli* (EPEC + EAEC + ETEC + STEC + *Shigella*/EIEC) accounted for 192 detections (45.7%). Not detected (0): *Vibrio* spp., *Vibrio cholerae*, *E. coli* O157, *Cyclospora cayetanensis*, *Entamoeba histolytica*. % of detections = target detections/420 total detections.

## 4. Discussion

### 4.1. Epidemiology of Acute Infections in the Salento Territory

The four panels together draw a coherent portrait of the microbiology of acute infections in the Lecce district. In the CNS compartment, the picture is overwhelmingly viral and led by enterovirus, in keeping with the established European epidemiology of aseptic meningitis, in which non-polio enteroviruses, particularly echovirus 30 and coxsackie/echovirus B-species, are the leading cause, with summer–autumn peaks and a marked predilection for infants and adults [11,12]. The substantial herpesvirus burden (HSV-1/2, VZV, and HHV-6) is also expected, although HHV-6 detection in CSF must be interpreted with caution, given frequent chromosomal integration and uncertain pathogenic significance [13]. The bacterial yield is minimal and almost entirely confined to *S. pneumoniae*, with only isolated cases of *H. influenzae*, *N. meningitidis* and *L. monocytogenes*, a profile that reflects the conjugate-vaccine era and the low background incidence of invasive bacterial meningitis in this population. The complete absence of *E. coli* K1 and *Cryptococcus* is consistent with a stream dominated by community-acquired, immunocompetent adult and paediatric presentations rather than neonatal or profoundly immunocompromised hosts. One corollary of a 90% all-negative rate deserves to be made explicit: part of that negativity may reflect aetiologies lying outside the panel altogether rather than the absence of infection. The ME panel interrogates a fixed list of fourteen targets and is blind, by design, to the arboviral agents of aseptic meningitis that are epidemiologically plausible in this territory, most notably Toscana virus, one of the leading causes of summer aseptic meningoencephalitis in central and southern Italy [14], and West Nile virus, which circulates seasonally in Italy and has repeatedly produced neuroinvasive disease during the vector season [15]; tick-borne encephalitis virus, by contrast, has its recognised Italian foci in the north-eastern regions and is not endemic in Salento, so its contribution here is expected to be negligible [16]. No systematic off-panel testing for these agents was performed during the study period, and we therefore cannot quantify their contribution to the negative fraction. That a proportion of the panel-negative meningitis presentations may have been caused by non-panel viruses, by bacteria attenuated by pre-analytical antibiotic exposure or present at low CSF volume, or by non-infectious conditions altogether, is an important qualification to the appropriateness argument developed below, and a strong argument for coupling any pleocytosis-gating rule with second-line reflex testing (Toscana virus, West Nile virus and broad-range 16S rRNA PCR) in panel-negative patients whose pleocytosis persists.

In the respiratory compartment, human rhinovirus/enterovirus dominates year-round, with adenovirus, influenza A, human metapneumovirus, and now-endemic SARS-CoV-2 completing the leading agents and the four seasonal coronaviruses circulating steadily beneath them. The apparent confinement of RSV to co-infections is epidemiologically unusual and most plausibly reflects the composition of the tested population (predominantly adults and mixed-presentation inpatients) and sampling/ordering patterns rather than a true biological feature; it should be read as a hypothesis-generating signal rather than a population estimate. The lower-airway ecology is, by contrast, classically polymicrobial and dominated by the organisms of severe community- and hospital-acquired pneumonia, with resistance determinants concentrated in Enterobacterales co-infections. The detection of KPC and, notably, a first NDM in the *K. pneumoniae* group mirrors the documented Italian trajectory from KPC endemicity towards emerging NDM-mediated carbapenem resistance, underscoring the value of local genotype surveillance [17].

In the enteric compartment, the panel describes a picture dominated by the diarrhoeagenic *Escherichia coli* pathotypes (chiefly EPEC and EAEC) and by *Clostridioides difficile* toxin, with *Salmonella* and *Campylobacter* as the principal classical bacterial enteropathogens and norovirus as the leading virus: a distribution typical of a mixed community and hospital population in a temperate Mediterranean setting. Two caveats temper interpretation. First, the high frequency of EPEC and EAEC, whose detection by multiplex PCR does not always equate to clinically significant disease, especially in adults and asymptomatic carriers. Second, *C. difficile* toxin-gene detection identifies carriage as well as infection and must be read against the clinical criteria for *C. difficile* infection rather than acted on in isolation [1,10,18]. The complete absence of *Vibrio*, *Cyclospora cayetanensis* and *Entamoeba histolytica* is consistent with the low local prevalence of these agents.

### 4.2. Diagnostic Appropriateness: A Setting-Specific Negativity Gradient

The most actionable finding is the appropriateness gradient. A 90% all-negative rate for the ME panel, stable across all three periods, signals that CSF syndromic testing is being ordered well beyond the population in whom it can plausibly yield a result. International experience is unambiguous on the remedy: Broadhurst and colleagues showed that restricting the ME panel to CSF with pleocytosis reduced utilisation by 42.7% and raised diagnostic yield by 61.8% (from 11.5% to 18.6%), while shortening empirical acyclovir exposure [4]; a French multicentre analysis found that a threshold of 10 leukocytes/mm^3^ avoided roughly two-thirds of inappropriate testing and improved agreement with clinical features, particularly for bacterial targets [19]; and additional cohorts have proposed formal decision rules to the same end [4,19]. Applied to our data, pleocytosis gating could reasonably be expected to avert on the order of 300 ME determinations across the study period while increasing yield towards 17–20%. The gastrointestinal panel mirrors this problem from a second angle: a 77.1% negativity rate, second only to the ME panel, marks another high-volume, low-yield stream, and here too the evidence-based levers are clear, since the FilmArray GI panel has limited utility in patients tested more than 72 h after admission (nosocomial diarrhoea, in which targeted *C. difficile* testing is preferable) and essentially none on short-interval repeats of an initial negative result, so confining it to community-onset diarrhoea, immunocompromised hosts and outbreak investigation (and blocking inpatient repeats) would raise yield and curtail expenditure with no loss of clinically actionable information [10,18]. The upper-respiratory panel, with 44% negativity, occupies an intermediate position amenable to tiered testing, whereas the pneumonia panel (27% negativity, 49% co-infection among positives) is being used appropriately, on a high-pre-test-probability, often critically ill population, and requires no restriction.

### 4.3. The Influenza-Subtyping Gap and Avian Influenza Surveillance

The 11 unsubtypeable or equivocal influenza-A determinations are individually rare but collectively important. Current commercial multiplex panels, including the BioFire Respiratory 2.1 panel, can detect influenza A (H5N1) through their generic influenza-A target but cannot assign the H5 subtype; validation work confirms reliable detection yet absent subtyping, which is why an integrated reflex H5 assay is recommended [5]. Public-health guidance is correspondingly explicit: any influenza-A–positive specimen that fails to return a valid seasonal subtype should be prioritised for referral to a public-health laboratory for H5/H7 testing and characterisation, and influenza-A–positive specimens from severely ill or epidemiologically exposed patients should be subtyped or sequenced [6]. The real-world value of this workflow is illustrated by the Los Angeles County investigation, in which BioFire-derived “unsubtypeable influenza A” results triggered CDC H5 reflex testing and uncovered human exposure linked to an infected animal [7]. In Salento, situated on a Mediterranean migratory flyway with backyard poultry and coastal wetlands, against a 2024–2026 background of expanding mammalian H5N1, the absence of any reflex sequencing for our equivocal influenza-A results is a concrete and avoidable blind spot. A single avian strain among those 11 specimens would have been reported and archived simply as “influenza A”.

### 4.4. Economic Considerations, High Negativity and Diagnostic Inappropriateness

Syndromic cartridges are costly consumables. Using the reagent prices applicable at our centre (€165 for each meningitis/encephalitis, upper-respiratory and gastrointestinal cartridge and €250 for each pneumonia cartridge), the 5381 determinations performed over 22 months represent a reagent expenditure of approximately €921,780 (Table 7). Of this, €535,360 (about 58% of the total spend, or roughly €292,000 per year) was consumed by the 3190 determinations that returned negative for every target. The imbalance is starkest for the ME panel (90% negativity; €113,025 on negative cerebrospinal fluid tests; €1652 per positive result) and, in absolute terms, for the two highest-volume low-yield streams: the upper-respiratory panel and the gastrointestinal panel, the latter alone accounting for €206,085 of spending on negative stool tests at a cost of €720 per positive result. A reagent-price sensitivity range of ±~15% (ME/RP/GI €140–190; PN €220–280) places the negative-result expenditure between approximately €455,000 and €616,000, leaving the conclusion unchanged in order of magnitude [20].

A test that is negative in nine of every ten cerebrospinal fluid requests, and in nearly half of upper-respiratory requests, is by definition being applied to a population in whom the pre-test probability does not justify it; the high negativity is therefore a direct, quantitative marker of diagnostic inappropriateness rather than of any failure of the assay, which performs well when correctly indicated. The corrective lever is consequently not the technology but the indication. The evidence is unambiguous that the single most effective measure is restricting CSF testing to patients with pleocytosis, which lowers utilisation by ~40% and raises yield by ~60% [4,19]; applied to our series, gating that averted ~300 ME determinations would save on the order of €50,000 over the study period (~€27,000 per year) while lifting yield from 10% towards 17–20%. A parallel restriction of the gastrointestinal panel, confining it to community-onset diarrhoea and blocking inpatient repeats and tests beyond 72 h of admission, would similarly remove a large share of its 1249 negative determinations and of the €206,085 they cost [10,18]. Realising this benefit, however, requires far more targeted protocols that are explicitly shared with and co-designed by the requesting clinicians (emergency medicine, neurology, infectious diseases and intensive care) rather than restrictions imposed unilaterally by the laboratory. Concretely, this means agreed clinical and laboratory gating criteria to select which patients are submitted to BioFire testing (for example, CSF-pleocytosis thresholds with explicit exemptions for neonates and immunocompromised hosts, and severity- and host-based criteria for respiratory testing), embedded in a structured request form with mandatory indication fields and soft or hard stops in the laboratory information system, and audited periodically against yield [1,2,20]. Such shared governance converts syndromic testing from a reflex, low-yield ordering habit into a deliberate, high-yield diagnostic act.

Framing the same figures in incremental rather than aggregate terms makes the stewardship case considerably more concrete (Table 8). Two metrics are informative. The first is the savings generated per unnecessary test avoided, which is simply the cartridge price (€165 for the ME, RP2.1 and GI panels, €250 for the pneumonia panel). The second is the marginal cost per additional positive result, that is, the expenditure absorbed by the lowest-yield stratum of a stream divided by the extra detections that stratum produces. Applying the parameters of the pleocytosis-gating study of Broadhurst and colleagues [4] to our ME series (a 42.7% reduction in volume with a 61.8% relative rise in yield) predicts 436 retained determinations and 325 averted, with yield rising from 10.0% to 16.2%; the averted stratum would have contained approximately five to six positives, so its implicit yield is about 1.7% and each additional positive detected within it costs on the order of €9700, against a reagent saving of €53,600 and a fall in the cost per positive from €1652 to roughly €1020 (−38%). For the gastrointestinal stream, where no local pleocytosis-equivalent gate exists, the same arithmetic can be run as a scenario: averting 20–40% of determinations (324–648 tests) would save €53,500–€106,900, and, at a residual yield of 3–5% in the averted stratum (inpatient testing beyond 72 h and short-interval repeats, for which the published yield is low [18]), each additional positive found there costs between roughly €3300 and €5500, four to seven times the current average cost per positive of €720. Put differently, the last euros spent in these two streams buy detections at five to ten times the price of the first, which is a workable operational definition of a stream that has outrun its indication. The pneumonia panel, at €340 per positive and 26.6% negativity, sits at the opposite end of the same scale and needs no restriction.

### 4.5. Beyond Cost: Qualitative Results, Semi-Quantitative Workarounds and the Case for Quantitative POCT

The limitations of the current platform are not solely economic; they are intrinsic to the nature of the data it returns. For the ME and upper-respiratory panels, the result is purely qualitative, with each target reported only as “detected” or “not detected”, and even the pneumonia panel offers only a coarse semi-quantitative read-out, binning bacterial nucleic acid into approximate categories (≈10^4^, 10^5^, 10^6^ or ≥10^7^ copies/mL) [8]. True quantification is therefore unavailable, and with it the ability to separate colonisation or contamination from infection, to gauge microbial load, or to monitor the response to treatment. To extract any quantitative information, the operator must resort to workarounds, most commonly inspecting the amplification/melt-curve peak (the height and shape of the fluorescence signal) as a surrogate for relative abundance. This “peak analysis” is, however, legitimate only under the assumption that amplification efficiency is identical across all targets, which is not guaranteed; in co-infections in particular, the relative quantity of each pathogen cannot be reliably established, and panel semi-quantitative values are known to run one to two logs higher than quantitative culture and to agree only moderately at low loads [8,9]. The clinical corollary is a high negative predictive value but a comparatively modest positive predictive value for detected targets and resistance genes, so a positive call must be correlated with culture and phenotype rather than acted upon in isolation [8]. These data-level constraints, compounded by the cartridge cost documented above, make it urgent to evaluate, after careful local validation against reference methods, complementary point-of-care platforms that deliver genuinely quantitative output (cycle-threshold or copy-number read-outs) at a lower unit cost, deployed selectively for the questions in which load and trend matter, while the broad qualitative syndromic panels are reserved for the high-pre-test-probability scenarios in which their breadth and speed are decisive [20,21].

### 4.6. International Comparison and Proposed Targeted Pathways

**Figure 4:**
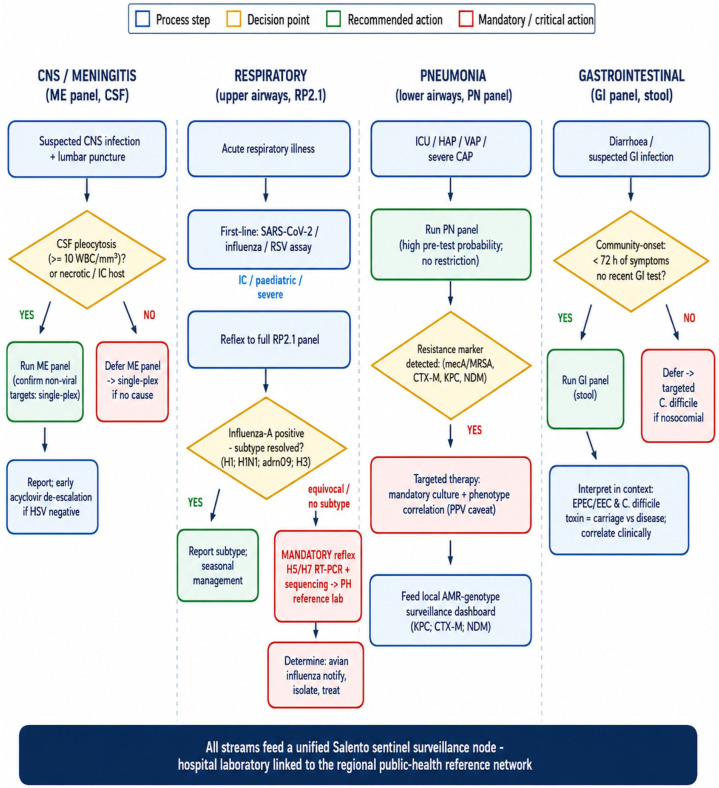
Proposed stewardship-based diagnostic algorithm for the four syndromic-testing streams at the ASL Lecce hub. The meningitis/encephalitis stream is gated on CSF pleocytosis (≥10 WBC/mm^3^), with an exemption pathway for neonates and immunocompromised hosts; the respiratory stream uses a first-line SARS-CoV-2/influenza/RSV assay reflexing to the full RP2.1 panel and, critically, mandatory reflex sequencing for every equivocal or unsubtypeable influenza-A result; the pneumonia stream is run without restriction in high-pre-test-probability patients, with obligatory culture/phenotype correlation of resistance markers feeding a local antimicrobial-resistance surveillance dashboard; the gastrointestinal stream is gated to community-onset diarrhoea and immunocompromised hosts, with hard stops on inpatient repeat testing and on testing beyond 72 h of admission (where standalone *C. difficile* testing is preferred). WBC, white blood cells; GI, gastrointestinal; IC, immunocompromised; PH, public health; PPV, positive predictive value; HAP/VAP, hospital-acquired/ventilator-associated pneumonia; CAP, community-acquired pneumonia.

Set against the international literature, our data reproduce a familiar pattern, high sensitivity and speed undermined by over-utilisation in the CNS setting, and point to the same evidence-based solutions [1,2]. We therefore propose, for the Lecce hub (Figure 4): (i) CSF-pleocytosis gating for the ME panel, with a defined exemption pathway for neonates and immunocompromised hosts and with targeted single-plex confirmation for non-viral targets [4,19]; (ii) a tiered respiratory algorithm in which a first-line SARS-CoV-2/influenza/RSV assay reflexes to the full panel for immunocompromised, paediatric or severe presentations, balancing yield against cost as advocated for flexible respiratory testing [21]; (iii) mandatory reflex sequencing of every equivocal or unsubtypeable influenza-A result, operationalised as a standing laboratory rule linking the hospital to the regional public-health reference network [5,6,7]; and (iv) systematic culture and phenotype correlation of pneumonia-panel resistance markers, feeding a local genotype surveillance dashboard for KPC/CTX-M/NDM [8,17]; and (v) gastrointestinal-panel gating to community-onset diarrhoea, immunocompromised hosts and outbreak investigation, with hard stops on inpatient repeats and on testing beyond 72 h of admission, where standalone *C. difficile* testing is preferred [10,18]. Such a programme would convert a high-volume, partially mis-targeted testing stream into a sentinel diagnostic node for the Salento territory.

### 4.7. Limitations

This study is retrospective and based on aggregate laboratory counts without linked clinical metadata; appropriateness is therefore inferred from yield by setting rather than from individual ordering indications, and CSF pleocytosis status, immune status and disease severity could not be incorporated. Culture and phenotypic correlation of molecular results, including resistance markers, were not available for this analysis. The study is, in this sense, purely molecular: panel targets are reported at species or pathotype level, so the *N. meningitidis*, *H. influenzae* and *S. pneumoniae* detections were neither serogrouped nor serotyped, and no inference about capsular epidemiology or vaccine coverage can be drawn from them. Aetiologies outside the fixed target lists, including Toscana virus, West Nile virus, and other arboviruses, in cerebrospinal fluid, were not investigated, so what we report is negativity for the panel targets and not proof of a non-infectious aetiology. Although duplicate determinations from the same patient were removed before analysis, the aggregate extraction carried no age, host-status or week-of-collection field, which is why the respiratory syncytial virus pattern and the unsubtypeable influenza-A cluster could not be stratified by paediatric or immunocompromised status or by season, and why the modelled savings in Table 8 are projections based on published gating parameters rather than observed outcomes of a local intervention. The influenza-subtyping denominator (2495 evaluable specimens) differs slightly from the full upper-respiratory cohort (2601), reflecting the subtyping-evaluable subset, and per-target prevalences are detection-based. Cost estimates are illustrative. Finally, this is a single-centre experience; although the catchment is well defined, generalisation beyond Salento should be cautious.

## 5. Conclusions

Twenty-two months of syndromic multiplex-PCR testing at the ASL Lecce microbiology hub draws a coherent picture of the local epidemiology of acute infections: a viral, enterovirus-led CNS compartment; a respiratory ecology dominated by rhinovirus/enterovirus; a polymicrobial, resistance-bearing lower-airway flora; and an enteric profile led by diarrhoeagenic *E. coli* and *Clostridioides difficile* toxin. Cutting across all four streams is a clear, setting-specific appropriateness gradient, whose two extremes—the 90% negativity of the ME panel and the 77% negativity of the gastrointestinal panel—are the prime targets for diagnostic stewardship: CSF-pleocytosis gating for the former and restriction of stool testing to community-onset diarrhoea for the latter, interventions shown elsewhere to curb volume and cost while raising yield. A second and distinct priority is surveillance: the 8% of influenza-A results left equivocal or unsubtypeable, none of which was referred for sequencing, is a small but genuine public-health blind spot for novel and avian influenza that a single standing reflex rule would close. Ultimately, directing the right panel to the right patient—and adding reflex sequencing and resistance-gene correlation where they matter—would turn this testing stream from a generator of negative results into an efficient sentinel surveillance system for the Salento territory.

## Figures and Tables

**Table 1:** Activity, positivity and negativity of the four syndromic panels by reporting period. Totals for the ME panel are derived from the sum of positive and negative determinations. This table reports activity and yield at the panel level only; per-target detections for each stream are given in Table 2, Table 3, Table 4, Table 5 and Table 6 and are deliberately not repeated here.

Panel	Tested	Single +	Co-inf.	Positive (%)	Negative (%)	Co-inf./pos.
ME (CSF)—total	761	76	0	76 (10.0)	685 (90.0)	0%
Upper resp.—total	2601	1183	268	1451 (55.8)	1150 (44.2)	18.5%
Pneumonia—total	399	150	143	293 (73.4)	106 (26.6)	48.8%
GI—total	1620	323	48	371 (22.9)	1249 (77.1)	12.9%
ALL PANELS	5381	1732	459	2191 (40.7)	3190 (59.3)	—

Single +, determinations positive for one target only; Co-inf., determinations positive for two or more targets in the same specimen; Positive, determinations with at least one target detected; Co-inf./pos., co-infections as a percentage of positive determinations. ME, meningitis/encephalitis panel; CSF, cerebrospinal fluid; Upper resp., upper-respiratory (RP2.1) panel; GI, gastrointestinal panel.

**Table 7:** Reagent-cost analysis of the four syndromic panels (1 July 2024–30 April 2026). Unit costs are the cartridge/reagent prices applied at the centre; totals exclude labour, instrument depreciation and overheads. Cost per positive = total panel cost divided by the number of positive determinations. ME, meningitis/encephalitis; CSF, cerebrospinal fluid; RP2.1, Respiratory 2.1 panel; PN, pneumonia panel; GI, gastrointestinal panel.

Panel	Unit Cost (€)	Tests (*n*)	Total Cost (€)	Negative (*n*)	Cost of Negatives (€)	Positives (*n*)	Cost per Positive (€)
ME (CSF)	165	761	125,565	685	113,025	76	1652
Upper resp. (RP2.1)	165	2601	429,165	1150	189,750	1451	296
Pneumonia (PN)	250	399	99,750	106	26,500	293	340
Gastrointestinal (GI)	165	1620	267,300	1249	206,085	371	720
Total/overall	—	5381	921,780	3190	535,360	2191	421

**Table 8:** Incremental economics of the proposed stewardship scenarios (1 July 2024–30 April 2026). Saving per avoided test equals the cartridge price. The marginal cost per additional positive is the reagent expenditure of the stratum that gating would remove, divided by the positives that stratum would have yielded; for the ME panel it is derived from the volume reduction and yield gain reported by Broadhurst et al. [4], for the GI panel from a 20–40% reduction scenario at an assumed residual yield of 3–5% in the removed stratum [10,18]. Figures are modelled projections, not observed outcomes, and exclude labour, instrument depreciation and overheads.

Panel	Cost per Positive, Current Practice (€)	Modelled Stewardship Scenario	Tests Averted (*n*)	Reagent Saving (€)	Marginal Cost per Additional Positive (€)
ME (CSF)	1652	CSF-pleocytosis gating (−42.7% volume, +61.8% relative yield) [4]	325	53,600	≈9700
Upper resp. (RP2.1)	296	Tiered first-line SARS-CoV-2/influenza/RSV assay reflexing to the full panel [21]	Not modelled	Not modelled	Not modelled
Pneumonia (PN)	340	None proposed (26.6% negativity; appropriate use)	—	—	—
Gastrointestinal (GI)	720	Gating to community-onset diarrhoea, hard stop beyond 72 h and on repeats (−20% to −40% scenario) [10,18]	324–648	53,500–106,900	≈3300–5500

## Data Availability

The aggregate data supporting the reported results are available from the corresponding author on reasonable request, subject to institutional data-governance approval.
